# Merkel cell carcinoma mimicking transformed chronic lymphocytic leukemia/small lymphocytic lymphoma

**DOI:** 10.1002/ccr3.2444

**Published:** 2019-09-26

**Authors:** Ismael Bah, Shaoying Li, C. Cameron Yin, Guilin Tang, Jie Xu

**Affiliations:** ^1^ Department of Hematopathology The University of Texas MD Anderson Cancer Center Houston TX

**Keywords:** chronic lymphocytic leukemia/small lymphocytic lymphoma, merkel cell carcinoma, PAX5, TdT

## Abstract

Although MCC has been reported in patients with CLL/SLL, it is extremely rare to observe these two within the same tumor. MCC's positivity for PAX5 and TdT may pose a diagnostic challenge by mimicking transformed CLL/SLL. A thorough workup is critical in reaching the correct diagnosis.

## Graphical abstract

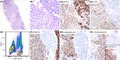

A 68‐year‐old man with a history of chronic lymphocytic leukemia/small lymphocytic lymphoma (CLL/SLL) presented with a rapidly enlarging neck mass. Core biopsy (Figure 1, panel A) displayed monotonous large neoplastic cells with round nuclei and fine chromatin (Figure 1, panel B, left) in the background of small lymphocytes (Figure 1, panel B, right). The large cells were positive for PAX5 (Figure 1, panel C) and TdT (Figure 1, panel D) and suspicious for large cell transformation of CLL/SLL. Flow cytometry revealed two abnormal populations: (a) a small population of small‐sized CD5+ lambda‐restricted B cells (panel E, red circle), consistent with CLL/SLL; (b) a large population of CD45‐negative large‐sized cells, which were CD56 + but negative for CD19, CD20, and CD22 (panel E, black circle), raising the concern for neuroendocrine tumor. Immunohistochemistry confirmed the large cells were positive for pancytokeratin, synaptophysin, chromogranin, and Merkel cell polyomavirus (MCPyV) (panels F‐I). He was diagnosed with Merkel cell carcinoma (MCC) in the background of CLL/SLL, involving salivary gland.

**Figure 1:**
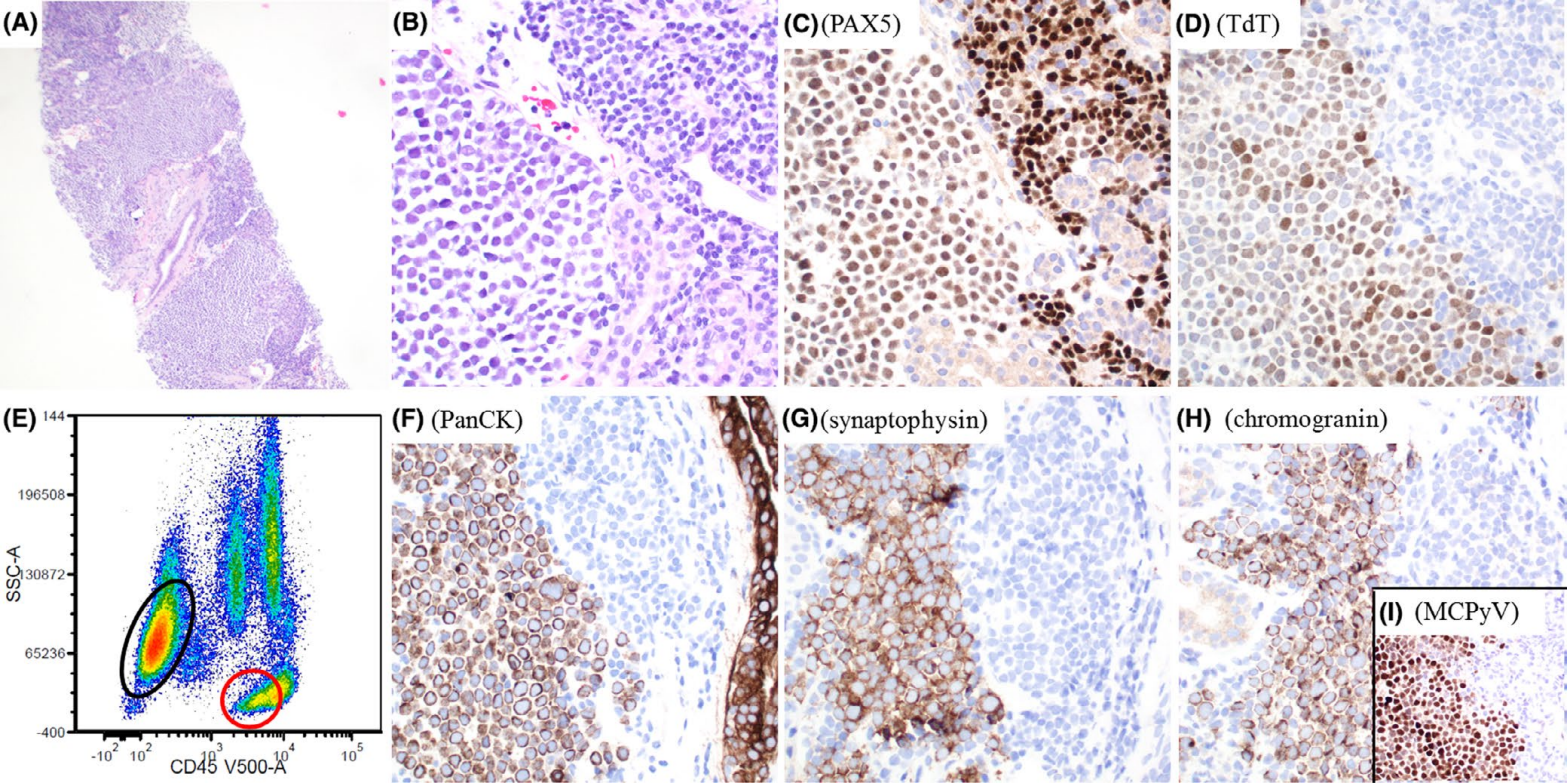
The large neoplastic cells showed fine chromatin (A; B, left), compared to the small lymphocytes with condensed chromatin (A; B, right). The large neoplastic cells were positive for PAX5 (C), TdT (D), pancytokeratin (F), synaptophysin (G), chromogranin (H), and Merkel cell polyomavirus (MCPyV) (I). E, Flow cytometry analysis showed a small population of CLL/SLL cells (red circle) and a large population of CD45− CD56+ large cells (black circle). A, hematoxylin and eosin stain, ×40. B, hematoxylin and eosin stain, ×400. C, D, F‐I, immunohistochemistry, ×400

Although MCC has been reported in patients with CLL/SLL,^1^ it is extremely rare to observe these two within the same tumor. MCC's positivity for PAX5 and TdT may pose a diagnostic challenge by mimicking transformed CLL/SLL. A thorough workup is critical in reaching the correct diagnosis.

## CONFLICT OF INTEREST

The authors declare that they have no conflicts of interest with the contents of this article.

## AUTHOR CONTRIBUTIONS

IB: collected data and drafted the manuscript. SL and CCY: helped making the pathological diagnosis. GT: revised the manuscript for intellectual content. JX: critically revised and finalized the manuscript.

## References

[1] Khezri F , Brewer JD , Weaver AL . Merkel cell carcinoma in the setting of chronic lymphocytic leukemia. Dermatol Surg. 2011;37(8):1100‐1105. 21631636 10.1111/j.1524-4725.2011.02045.x

